# Effect of fluoride on major organs with the different time of exposure in rats

**DOI:** 10.1186/s12199-018-0707-2

**Published:** 2018-05-16

**Authors:** Thanusha Perera, Shirani Ranasinghe, Neil Alles, Roshitha Waduge

**Affiliations:** 1 Postgraduate Institute of Science, University of Peradeniya, Peradeniya, Sri Lanka; 2 Department of Biochemistry, Faculty of Medicine, University of Peradeniya, Peradeniya, Sri Lanka; 3 Department of Pathology, Faculty of Medicine, University of Peradeniya, Peradeniya, Sri Lanka

**Keywords:** Drinking water, Fluoride toxicity, Chronic kidney disease of unknown etiology (CKDu), Sri Lanka

## Abstract

**Background:**

High fluoride levels in drinking water in relation to the prevalence of chronic kidney disease of unknown etiology (CKDu) in Sri Lanka were investigated using rats as an experimental model.

**Method:**

The effects of fluoride after oral administration of Sodium fluoride (NaF) at levels of 0, 0.5, 5 and 20 ppm F^−^ were evaluated in adult male Wistar rats. Thirty-six rats were randomly divided into 4 groups (*n* = 9), namely, control, test I, II, and III. Control group was given daily 1 ml/rat of distilled water and test groups I, II, and III were treated 1 ml/rat of NaF doses of 0.5, 5, and 20 ppm, respectively, by using a stomach tube. Three rats from the control group and each experimental group were sacrificed after 15, 30, and 60 days following treatment. Serological and histopathological investigations were carried out using blood, kidney, and liver.

**Results:**

No significant differences were observed in body weight gain and relative organ weights of the liver and kidney in fluoride-treated groups compared to control group. After 60 days of fluoride administration, group I showed a mild portal inflammation with lytic necrosis while multiple areas of focal necrosis and various degrees of portal inflammation were observed in groups II and III. This was further confirmed by increased serum aspartate aminotransferase (AST), alanine aminotransferase (ALT), and alkaline phosphatase (ALP) activities. As compared with control and other treated groups, group III showed a significantly higher serum AST activity (*p* < 0.05) and ALT activity (*p* < 0.05) after 60 days and ALP activity with a significant difference (*p* < 0.05) after 15, 30, and 60 days. The renal histological analysis showed normal histological features in all groups with the elevated serum creatinine levels in group III compared to those in the groups I and II (*p* < 0.05) after 60 days. Significantly elevated serum fluoride levels were observed in group II of 30 and 60 days and group III after 15, 30, and 60 days with respective to control groups (*p* < 0.05).

**Conclusion:**

Taken together, these findings indicate that there can be some alterations in liver enzyme activities at early stages of fluoride intoxication followed by renal damage.

## Background

Chronic kidney disease (CKD) which is recognized as progressively deterioration of renal function has become a growing global health crisis [1]. CKD is commonly found in the countries like USA, Canada, Iran, and many Asian countries such as Korea, Taiwan, Japan, China, India including Sri Lanka as a consequence of diseases such as diabetes and hypertension [2, 3]. However, Since the 1990s, a severe form of CKD, which is not attributable to these common causes but suspected to be environmental factors, have been reported in Sri Lanka, and thus it is termed as chronic kidney disease of unknown etiology (CKDu) [4, 5]. This rising disease is slowly progressive and asymptomatic until late stages; hence, this makes diagnosis and treatment complicated [6]. Many people in the north central province (NCP) of Sri Lanka have died from CKDu, and the disease is now spreading to neighboring districts in the north western, eastern, and Uva provinces [7, 8]. Reports reveal that around 400,000 people in north central region of Sri Lanka may be affected by this chronic kidney disease [9].

Ground-water which is the main source of drinking water for people in CKDu endemic regions and the geochemistry of groundwater in such regions is marked by the presence of fluoride in significant quantities, varies from 2 to 5 ppm. According to Chandrajith et al., the maximum fluoride levels in endemic CKDu areas of Girandurukottee, Nikawewa, Medawachchiya, and Padawiya were 2.14, 5.3, 4.9, and 1.33 ppm, respectively [10]. This is much greater than the 0.5 ppm, the maximum acceptable concentration of fluoride ions in drinking water for tropical countries by the World Health Organization (WHO) [11]. Not only in Sri Lanka, but also in other countries such as India, Pakistan, China, and Kenya, fluoride contamination in ground water is a serious issue since the South Asia considered as the epicenter of fluoride contamination in ground water [12]. According to Ali (2006), almost all the states in India, East Punjab area of Pakistan and Liaodong mountains, and Liaoning Peninsula, hills of south eastern China, were reported to exceed WHO standard of fluoride [12].

Many studies have shown that exposed to high fluoride concentrations in drinking water elevated the levels of renal and liver function enzymes in serum and cause severe histological changes of the liver and kidneys [13–16]. In study done by Guo and Sun (2003), NaF-treated Wistar rats with 50, 100, and 150 mg NaF/L with their drinking water for 3 months have shown significant increase of serum glutamic pyruvic transaminase (SGPT) and serum glutamic oxaloacetic transaminase (SGOT) activities and hepatic damages [13]. In another study, chicken were exposed to 10, 20, and 30 mg/g of NaF on weekly basis for 4 weeks have elevated the levels of the liver function indicators, alanine aminotransferase (ALT), aspartate aminotransferase (AST), and alkaline phosphatase (ALP) [15]. Mean time such studies have shown that fluoride has deleterious effects on kidneys exposed to high concentrations (50, 100, 125,150, 250 ppm, etc.) of fluoride [14–16]. According to Zhan et al., pigs exposed to fluoride concentrations of 100 and 250 mg/Kg were showed significantly increased serum creatinine and urea levels and deleterious effect to kidney structure and function [16].

Therefore, it is very important to understand how the reported fluoride levels in the endemic areas in Sri Lanka can alter serological parameters and cause gross and microarchitectural changes in kidney and liver in rats. But there are few studies have done to find the effects of fluoride on multiple organs (especially both kidney and liver) with the low levels of exposure. And also, it is very important to find out the damages caused by the reported fluoride levels in drinking water in CKDu endemic areas in Sri Lanka to the kidney and/or liver. Therefore, in this study we used three concentration, 0.5, 5, and 20 ppm which were the WHO recommended health-related value for fluoride ions for tropical countries, commonly reported high fluoride level in CKDu endemic areas based on the geological data and to represent a high fluoride concentration used in other similar studies, respectively. And here, we examined the effect of fluoride levels at different concentration for variable time period on kidney and liver in rats using serological parameters and histopathological lesions as tools to correlate with CKDu.

## Methods

Experiment was carried out with healthy 36 male Wistar rats (origin; Clea Japan, Inc.) purchased from Medical Research Institute, Sri Lanka, weighing 134.22 ± 9.92 g and aged 4–5 weeks. They were reared under sanitary conditions and maintained at 25 °C and 50% humidity on a 12-h light/dark cycle. The rats were acclimated for 1 week in a housing facility and randomly divided into four groups (control, group 1, II, and III) each with nine rats. They were provided with ad libitum rat feed and water. Ethical approval for the experimental procedure was obtained from the Ethics Committee of Postgraduate institute of science, University of Peradeniya, Sri Lanka, and the animal experiments were carried out according to the International Guiding Principles for Biomedical Research Involving Animals (Council for the International Organizations of Medical Sciences 2012) [17].

### Treatment and sampling

Their body weights were recorded prior to fluoride treatments and considered as initial body weights. Control group was given 1 ml/day/rat of distilled water while group I, II, and III were given sodium fluoride (NaF) (Sigma, Missouri, USA) dissolved in distilled water at a dose of 0.5, 5, or 20 ppm/1 ml/day/rat, respectively. In all cases, administrations of the fluoride or distilled water were carried out using a stomach tube to ensure precise and accurate dosing of animals [18]. In addition, all of the rats had free access to food and water. Three rats from the control and each experimental group I, II, and III were sacrificed after 15, 30, and 60 days. Body weight of each rat was recorded as final body weight and blood from each animal was withdrawn before sacrificed. Liver and kidneys were dissected out carefully and their fresh weights were recorded.

### Determination of body weight gain and relative organ weights

Weight gain of rats was calculated as the difference between final and initial body weights. Relative organ weights were determined by calculating ratio between fresh organ weight and body weight (organ weight/body weight %).

### Histological examination

Kidney and liver samples for histopathology were collected and fixed in 10% phosphate-buffered formalin for 24 h. Thereafter, the tissues were dehydrated in graded concentrations of xylene, embedded in molten paraffin wax, and sectioned at 5 μm. The sectioned tissues were fixed on grease-free glass slides and stained with hematoxylin and eosin (H&E) [19]. Finally, they were observed under a light microscope by two pathologists independently. The scoring was done to increase the sensitivity according to Knodell histological activity index/scoring (HAI) system [20]. Briefly, this was based on the assessment of portal/periportal activity with or without bridging necrosis, intralobular necrosis, portal inflammation, and fibrosis. In Knodell scoring, the scores for periportal/bridging necrosis (0–10), intralobular degeneration (0–4), portal inflammation (0–4), and fibrosis (0–4) were combined to derive the total HAI score (range = 0–22). Finally levels were graded as follows: 0 = no inflammation, 1–4 = minimal inflammation, 5–8 = mild inflammation, 9–12 = moderate inflammation, and 13–18 = marked inflammation.

### Biochemical assays

Serum was obtained by centrifuging at 5000 rpm for 5 min, and biochemical assays were carried out for serum creatinine (Agappe, Kerala, India), uric acid (Agappe, Kerala, India), aspartate aminotransferase (AST, Randox, Crumlin, UK), alanine aminotransferase (ALT, Agappe, Kerala, India), and alkaline phosphatase (ALP, Fortress, Northern Ireland, UK) using commercially available assay kits. The absorbances were measured using a spectrophotometer (Shimadzu, Kyoto, Japan).

### Determination of fluoride levels in serum

Fluoride in serum was measured with fluoride ion selective electrode (Thermo Scientific Orion, USA).

### Statistical analysis

All the data were presented as mean ± SD and were analyzed using one-way analysis of variance (ANOVA) and Fisher’s multiple comparison tests. Results were declared significant at *p* < 0.05.

## Results

### No significant changes were observed in body weight gain in fluoride-treated groups

As an indicator of toxicity, first, we measure the changes in body weight gain of rats after 15, 30, and 60 days were calculated as mentioned in the methodology section. There were no significant differences in body weight gain when compared the fluoride-treated groups I, II, and III with their respective control groups after 15, 30, and 60 days (Table 1).

**Table 1:** Comparison of body weight gain of rats exposed to 0, 0.5, 5 and 20 ppm fluoride for 15, 30 and 60 days. Data are expressed as mean ± S.D., (*n* = 3)

Groups	Body weight gain (g)		
	15 days	30 days	60 days
Control	24.00 ± 4.58	76.00 ± 8.54	124.67 ± 2.08
Group I (0.5 ppm)	19.33 ± 4.04	70.67 ± 15.50	127.33 ± 9.29
Group II (5 ppm)	24.67 ± 7.23	62.67 ± 7.51	126.00 ± 11.53
Group III (20 ppm)	23.33 ± 4.51	66.67 ± 11.02	129.00 ± 5.00

### Relative organ weights are not affected with the fluoride treatment

**Fig. 1:**
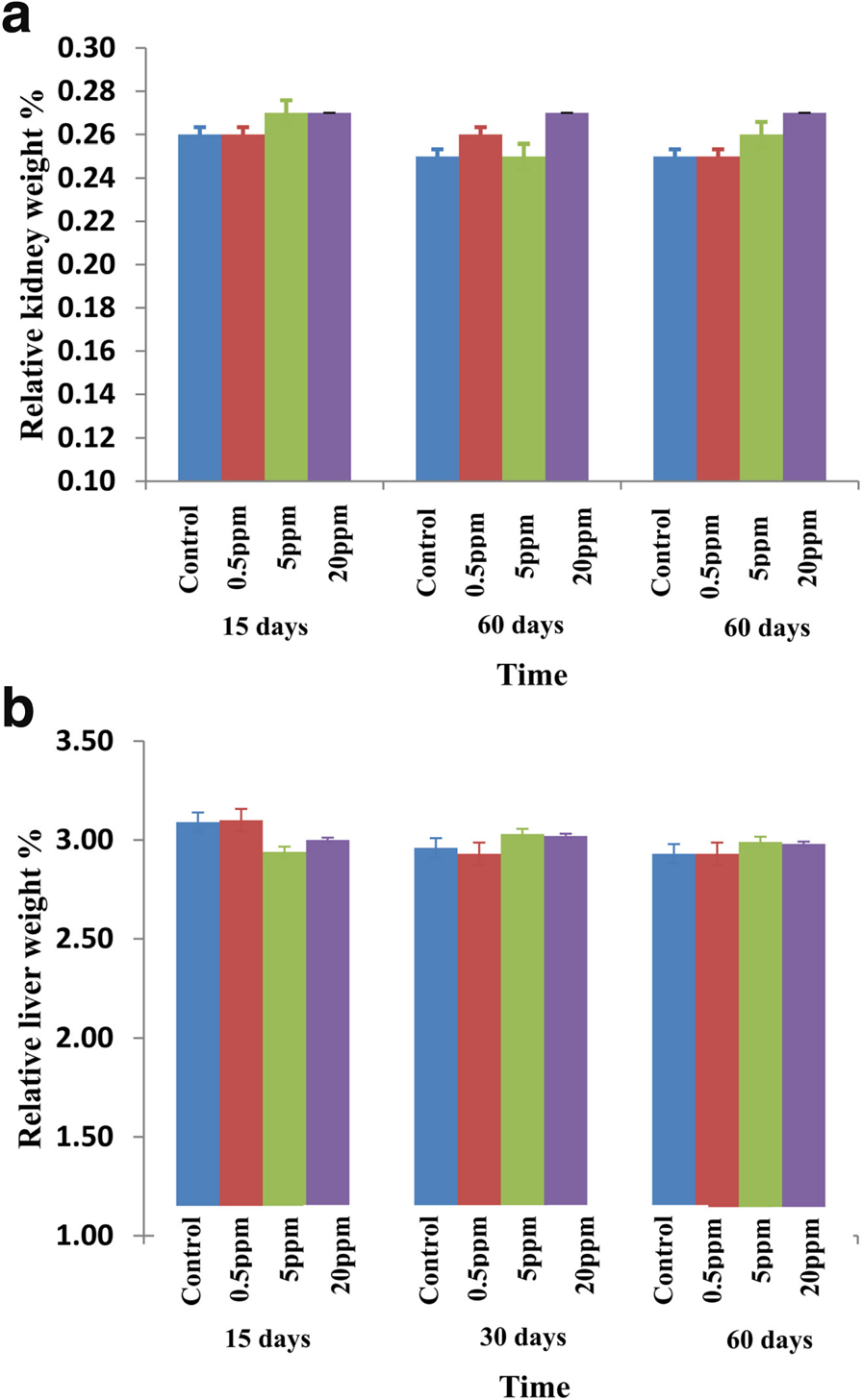
Relative organ weights of rats treated with fluoride. **a** Relative kidney weights. **b** Relative liver weights of rats exposed to distilled water (control) and different concentrations of fluoride (0.5, 5, and 20 ppm) for 15, 30, and 60 days. Error bars represent standard error

Relative organ weights were calculated as an important requirement to see the ability to assess the effect of xenobiotics on specific organs between treated and untreated (control) groups. Therefore, the fresh weights of livers and kidneys collected from rats were analyzed for relative organ weights as mentioned in the methodology (Fig. 1). There were no significant difference in the relative kidney weights of rats treated with fluoride at different concentrations, 0.5, 5, and 20 ppm compared to control group for 15, 30, and 60 days (Fig. 1a). The differences were also not significant in the relative liver weights of rats between fluoride-treated groups and their respective control groups after 15, 30, and 60 days (Fig. 1b).

### Fluoride induced varies degrees of portal inflammation and focal necrosis on hepatic histology resulting in mild hepatic inflammation

**Fig. 2:**
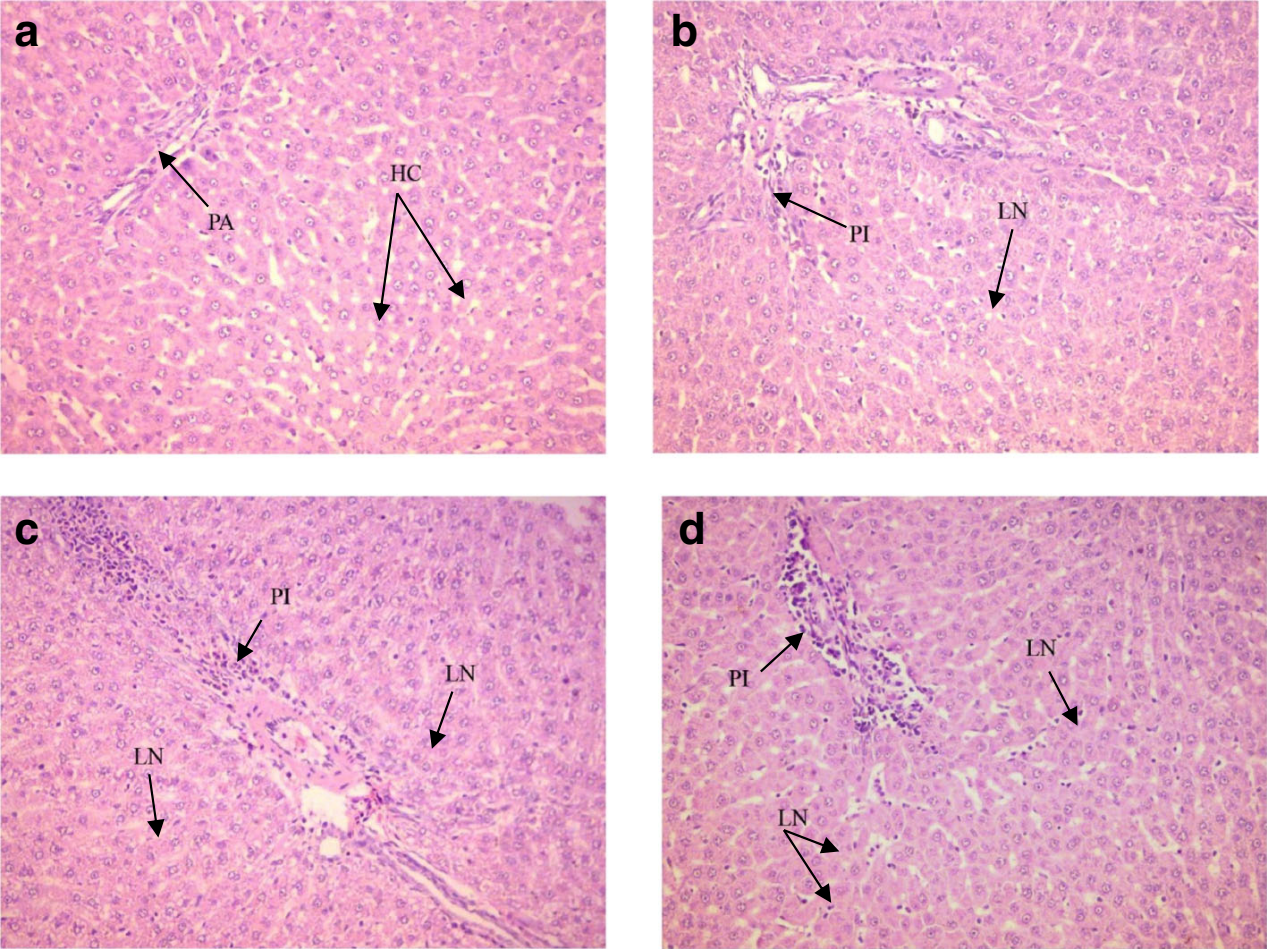
Histopathological changes of liver tissues stained with Hemotoxylin and Eosin (HE) for 60 days (X200); (*N* = 3/group). **a** Control, normal liver tissue with no abnormalities portal area (PA), and hepatocytes (HC). **b** 0.5 ppm NaF, inflammatory cells in the portal triads (PI) at moderate level and lytic necrotic (LN) hepatocytes. **c** 5 ppm NaF, inflammatory cells in the portal triads (PI) at moderate levels, and widespread necrosis (LN). **d** 20 ppm NaF, inflammatory cells in the portal triads (PI) at moderate levels, and multifocal lytic necrosis (LN)

Then, histopathological examinations in hepatic and renal tissues were performed in order to find out any tissue damages due to fluoride exposure. Histological sections of hepatic tissues were showed no inflammation in control groups while fluoride administrated groups indicated variable histological changes (Table 2). After 15 days of fluoride administration, groups II and III showed few focal areas of necrosis of hepatic cells and mild portal inflammation in hepatic tissues whereas the intensity of these focal necrosis and the inflammatory cells in the portal areas were remarkably increased with the exposure time of 30 days. After 60 days of administration, group I showed a mild portal inflammation with lytic necrosis while multiple areas of focal necrosis and various degrees of portal inflammation appeared in groups II and III (Fig. 2). To confirm these observations, Knodell histological scoring was carried out in order to obtain a quantitative assessment of liver inflammation and damages. These data showed minimal inflammation in groups II and III at 15 days and group I at 60 days. Moreover, mild liver inflammation was obtained in groups II and III after 60 days of fluoride administration with a maximum score of 6.

**Table 2:** Histopathological changes of hepatic cells in rats exposure to different concentrations of fluoride

Groups		Portal inflammation	Lytic necrosis	Knodell score^a^	Interpretation
15 days	Control	0	0	0	No inflammation
	0.5 ppm	0	0	0	No inflammation
	5 ppm	1	1	2	Minimal inflammation
	20 ppm	1	1	2	Minimal inflammation
30 days	Control	0	0	0	No inflammation
	0.5 ppm	0	1	1	Minimal inflammation
	5 ppm	1	1	2	Minimal inflammation
	20 ppm	1	3	4	Minimal inflammation
60 days	Control	0	0	0	No inflammation
	0.5 ppm	1	3	4	Minimal inflammation
	5 ppm	3	3	6	Mild inflammation
	20 ppm	3	3	6	Mild inflammation
Portal inflammation: 0, no portal inflammation, 1, mild (sprinkling of inflammatory cells in < 1/3 of portal tracts), 3, moderate (increased inflammatory cells in 1/3–2/3 of portal tracts), 4, marked (dense packing of inflammatory cells in > 2/3 of portal tracts).				Necrosis: 0, none, 1, mild (acidophilic bodies, ballooning degeneration, and/ or scattered foci of hepatocellular necrosis in < 1/3 of lobules or nodules) 3, moderate (involvement of 1/3–2/3 of lobules or nodules) 4, marked (involvement of > 2/3 of lobules or nodules)	

0 = no inflammation, 1–4 = minimal inflammation, 5–8 = mild inflammation, 9–12 = moderate inflammation and 13–18 = marked inflammation

^a^Knodell HAI score is the combined scores for periportal necrosis, intralobular necrosis, portal inflammation, and fibrosis to yield a possible range of 0–22. As used in clinical trials, the first three categories are often totaled to give a necroinflammatory score (0–18), while the fibrosis score (0–4) is reported separately

Though there were hepatic histopathological changes in rats varied with the concentration of fluoride, the results of the renal histological analysis showed normal histological features in all groups (data not shown).

### The serum creatinine levels were increased in group III after 60 days

**Fig. 3:**
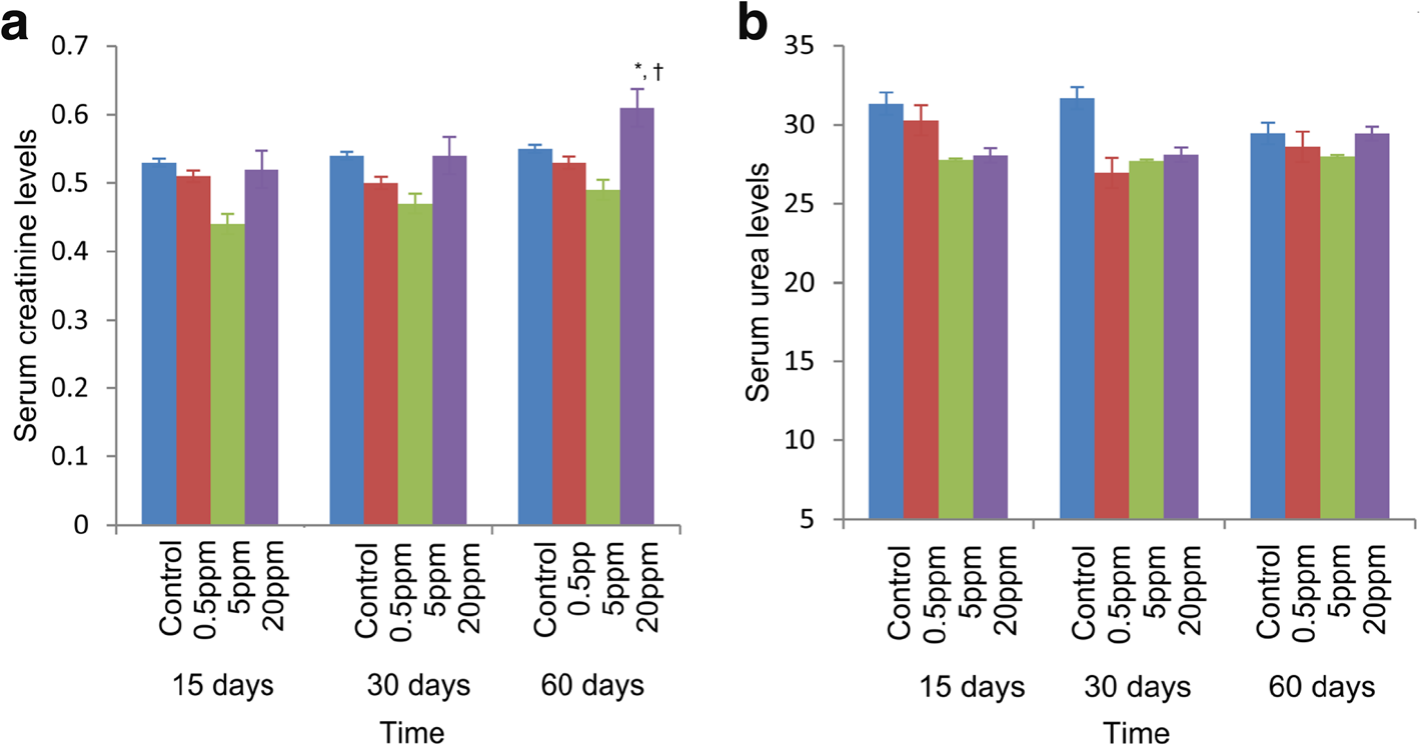
Effects of fluoride on serum creatinine and urea in rats after 15, 30, and 60 days. **a** Serum creatinine levels. **b** Serum urea levels of rats exposed to distilled water and different concentrations of fluoride (0.5, 5, and 20 ppm) for 15, 30, and 60 days. *p < 0.05 compared with 0.5 ppm; †*p* < 0.05 compared with 5 ppm. Error bars represent standard error

In order to clarify the results of histopathological changes, serum creatinine and urea levels were measured as markers of renal cellular functions (Fig. 3). Creatinine levels in all test groups were not significant with their respective control groups after 15 and 30 days. However, the creatinin levels in group III had a significantly higher mean value compared to those in the groups I and II (*p* < 0.05) after 60 days (Fig. 3a). In contrast, no significant differences were noted for serum urea concentrations among the groups I, II, and III with their control groups after 15, 30, and 60 days (Fig. 3b).

### Enzyme activity of serum AST, ALP, and ALT levels were higher in all groups received the highest dose (20 ppm) of fluoride

**Fig. 4:**
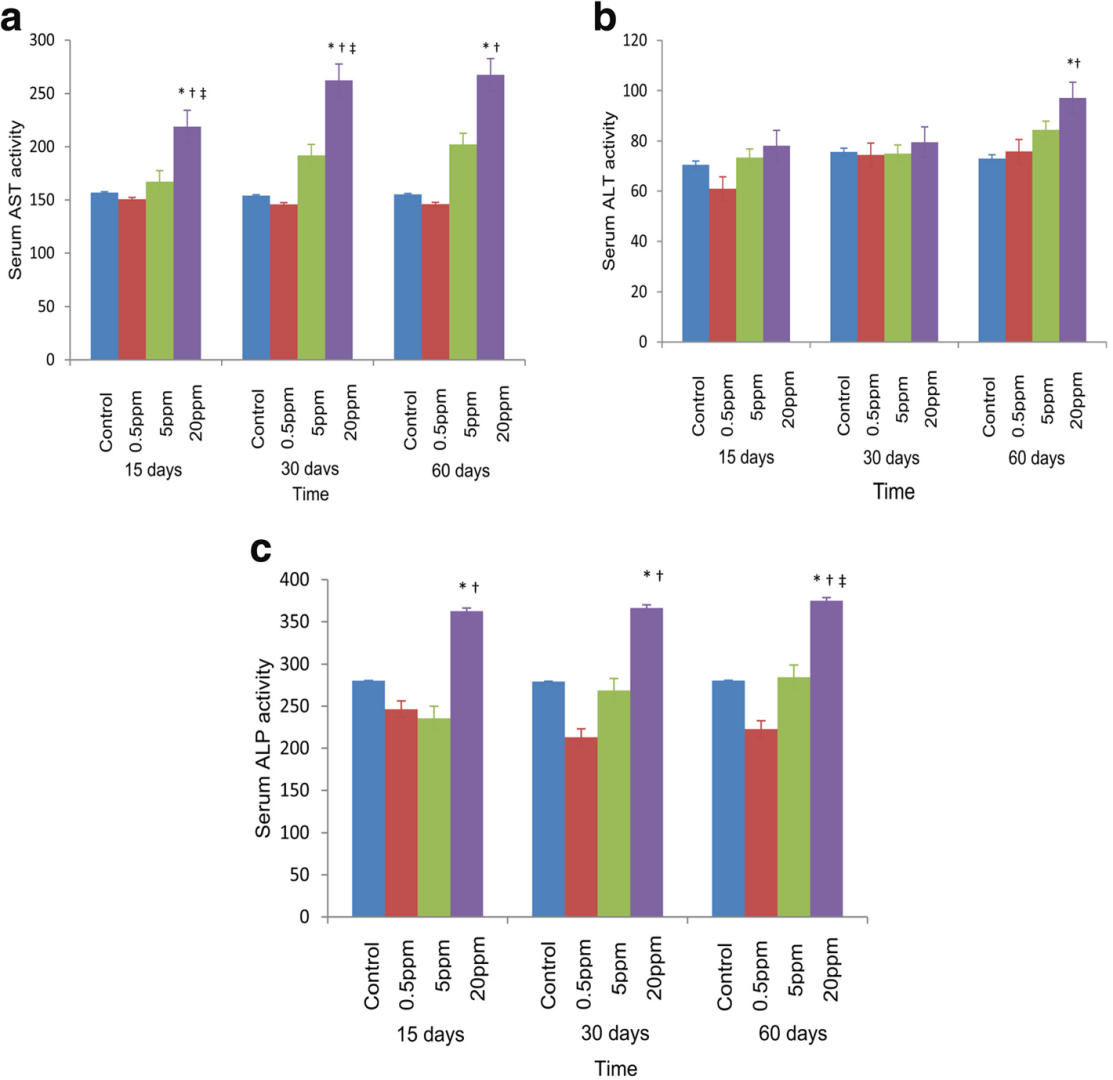
Effects of fluoride on the activity of serum aspartate aminotransferase (AST), alkaline phosphatase (ALP), and alanine aminotransferase (ALT) in rats after 15, 30, and 60 days. **a** Effects of fluoride on the activity of serum aspartate aminotransferase (AST) in rats after 15, 30, and 60 days. *p < 0.05 compared with control, †*p* < 0.05 compared with 0.5 ppm, ‡*p* < 0.05 compared with 5 ppm. **b** Effects of fluoride on the activity of serum alanine aminotransferase (ALT) in rats after 15, 30, and 60 days. *p < 0.05 compared with control, †*p* < 0.05 compared with 0.5 ppm. **c** Effects of fluoride on the activity of serum alkaline phosphatase (ALP) in rats after 15, 30, and 60 days. *p < 0.05 compared with 0.5 ppm, †*p* < 0.05 compared with 5 ppm, ‡*p* < 0.05 compared with control. Error bars represent standard error

The activity of serum aspartate aminotransferase (AST), alkaline phosphatase (ALP), and alanine aminotransferase (ALT) were measured as the liver function tests in rats after different concentrations of fluoride exposure (Fig. 4). The serum AST activity was higher in those groups receiving the highest dose (20 ppm) of fluoride. After 15, 30, and 60 days of fluoride exposure, group III showed a significantly higher serum AST activity (*p* < 0.05) compared to the control and other test groups I (*p* < 0.05) and II (*p* < 0.05) (Fig. 4a). There were no significant differences in serum ALT activity between control and fluoride-treated groups I, II, and III after 15 and 30 days while ALT activity in group III was significantly increased (*p* < 0.05) compared to the control and group I within 60 days (Fig. 4b). The highest values of serum ALP activities were observed in group III with a significant increase (Fig. 4c) and the difference was statistically significant with control, groups I, II at 15, 30, and 60 days (*p* < 0.05).

### High fluoride intake alters the serum fluoride level

Serum fluoride concentration is recognized as indicator for fluoride exposure [21]. Therefore, we examined the serum fluoride levels and they were range from 0.06 to 0.07 in control and other experimental groups after 15, 30, and 60 days (Table 3). Group I had no significant difference in fluoride levels in serum after 15, 30, and 60 days compared to control groups. Though the serum fluoride concentrations were not significantly different between the control, group I, and group II of 15 days, prolonged exposure resulted statistically significant higher serum fluoride levels in group II of 30 and 60 days and the high dose-treated group III after 15, 30, and 60 days with respective to control groups (*p* < 0.05).

**Table 3:** Serum fluoride levels in rats after 15, 30, and 60 days

Groups	15 days	30 days	60 days
Control	0.060 ± 0.003	0.060 ± 0.002	0.060 ± 0.001
Group I (0.5 ppm)	0.062 ± 0.001	0.062 ± 0.001	0.062 ± 0.003
Group II (5 ppm)	0.062 ± 0.001	0.065 ± 0.003*	0.067 ± 0.001*
Group III (20 ppm)	0.063 ± 0.002*	0.066 ± 0.001*	0.07 ± 0.003*

Values are mean ± SD (*n* = 3)

*p < 0.05 compared with control

## Discussion

According to the results, there are no significant differences in body weight gain and relative organ weights changes among the groups. The evaluation of relative organ weight changes have been used to detect the effects of chemically stimulated changes to an organ either by oral or inhalation routes in the evaluation of toxicological studies [22]. Further, it can be used to assess whether an organ or tissue were subjected to any damages or not [23]. The results reveal that within the dose range used, fluoride has no effect on relative kidney and liver organ weights up to 60 days, compared to the control group. Similar results had been found in a study carried out by Tsunoda [8], the mice were given NaF dissolved in distilled water at the concentrations of 0, 1, 5, 25, and 125 ppm F ion in their drinking water for 1 month and were not seen any significant differences in body weights and relative organ weights [14].

Liver is an important organ for metabolism and detoxification of foreign substances [24]. In the present study, liver histopathological changes varied with the concentration of fluoride in treated groups with respect to the control groups. Histopathological changes logical sectioning which indicated various degrees of hepatocellular necrosis and portal inflammation in the treated groups. Similar results were observed in the liver cells of animals exposed to 25 mg/kg F for 4 weeks [25]. Evidences of changes in liver may relate to that the liver has a central role as a detoxifying organ towards xenobiotics and chemicals [24]. The toxicants have been revealed by abnormal metabolic functions, reduced activity of detoxification reaction, and altered structure of sub cellular organelles [26]. According to the Thangapandiyan and Miltonprabu (2014), these pathological alterations in the fluoride-treated liver tissues could be due to the accumulation of free radicals by fluoride ions [25]. Diagnostic evaluation of liver tissue is largely based on a thorough examination of sections stained with hematoxylin and eosin (H&E), and it has been the most universal and traditional method for examination of formalin-fixed, paraffin-embedded tissue sections [27]. The inflammatory cells could be lymphocytes, plasma cells, or macrophages, and they were stained as mononuclear inflammatory cells with H&E stain. Therefore, additional special stains such as immunohistochemical stains may be useful to highlight or identify features that are not easily seen on an H&E stain [28].

The transverse section of kidneys exposed to 0.5, 5, and 20 ppm F has shown no histological structure changes. There were many contradictory reports regarding F-induced toxicity in kidney with high concentrations. Zhan indicated that supplemental fluoride treatment (100 and 250 mg kg^− 1^) caused severe renal histological changes as well as increased renal cell apoptosis [16]. According to Tsunoda, 125 mg l^− 1^ F concentration group significantly increased the concentrations of fluoride in the liver and kidney compared to the control [14]. Though there are many studies carried out with high fluoride concentrations, studies on concentrations comparable to the existing environmental fluoride concentrations are, however, relatively limited.

Serum creatinine concentrations are widely used clinically as an index of renal function [29]. Increases in serum creatinine level in group III (20 ppm) is an indication of reduced clearance of the substances in turn the impairment of renal function of treated groups. In particular, the urea concentration is determined by the balance of urea synthesis and excretion by the kidneys. There is no significant difference in serum urea levels among the test groups compared to the control up to 60 days. Even though the level of creatinine in serum and blood urea are considered as established markers for kidney function, serum creatinine is a more sensitive indicator, as many extra renal conditions such as dehydration can alter urea levels [30].

Further, the aminotransferases are important diagnostic and prognostic tools of liver disease and a specific indication of the impairment of liver functions [31]. Elevated levels of AST, ALT, and ALP levels in those groups receiving the highest dose of fluoride showed the impairment of liver functions. The AST and ALT levels are increased to some extent in almost all liver diseases [32], and similar results have been reported in animal experiments and in fluorotic children. Further, more pronounced levels of AST, ALT, and ALP were reported in the chicks exposed to high fluoride [15]. According to Shivashankara [33], there was a significant increase in serum levels of ALP, ALT, and AST on fluorotic children whereas creatinine was significantly increased and urea levels were markedly reduced indicating disturbed liver function.

Serum fluoride concentration is recognized as a good indicator of fluoride exposure [21]. The detection of ionic form of fluoride, by the ion-specific electrode, is common in dentistry, medicine, and public health. In the present study, serum fluoride levels in the high dose-treated groups (group III) were significantly greater (*p* < 0.05) than the control group. Similar findings have also been reported in rats due to exposure to fluoride. Their results revealed that the fluoride content in urine and serum in the three experimental groups was significantly higher than in the control group (*p* < 0.01) in a dose-dependent manner [13]. A study from Xiang [22] found that there was a significant difference between the serum fluoride levels and the drinking water fluoride concentrations of two villages, Wamiao (2.18 ± 0.86 ppm) and Xinhuai (0.37 ± 0.09 ppm). Another case-controlled study has compared urinary, blood plasma, and serum fluoride levels of people living in endemic areas of the Thar Desert, Sindh, Pakistan, consuming groundwater with fluoride concentrations as high as 4.00–10.00 mg/L with those consuming groundwater with low fluoride levels of 0.30 mg/L, and there was a highly significant difference (*p* < 0.001) between the serum fluoride levels in endemic areas compared to control [34].

## Conclusions

Fluoride exposure impaired hepatocytes and hepatic function, which was strongly supported by the necrosis and portal inflammation histopathologically and increased serum AST, ALT, and ALP activities. Further, it has been demonstrated that there is a possibility of inducing renal damage by high fluoride levels for longer period of administration due to elevated creatinine levels. Taken together, the fact of the present early changes of liver functions following fluoride exposure before the renal lesions are appeared can be further taken into consideration by the health sector during screening of individuals in CKDu prevalent areas.

## Abbreviations

ALP: Alkaline phosphatase
ALT: Alanine aminotransferase
ANOVA: Analysis of variance
AST: Aspartate aminotransferase
CKDu: Chronic kidney disease of unknown etiology
F: Fluoride
HE: Hematoxylin and eosin
NaF: Sodium fluoride
NCP: North central province
WHO: World Health Organization
